# Degradation of Chemical Warfare Agent Nitrogen Mustard Using Ferrate (VI)

**DOI:** 10.3390/toxics11070559

**Published:** 2023-06-26

**Authors:** Miroslav Labaška, Miroslav Gál, Tomáš Mackuľak

**Affiliations:** 1 Department of Environmental Engineering, Faculty of Chemical and Food Technology, Slovak University of Technology in Bratislava, Radlinského 9, 812 37 Bratislava, Slovakia; tomas.mackulak@stuba.sk; 2 Department of Inorganic Technology, Faculty of Chemical and Food Technology, Slovak University of Technology in Bratislava, Radlinského 9, 812 37 Bratislava, Slovakia

**Keywords:** chemical warfare agent, decontamination, nitrogen mustard, ferrate (VI)

## Abstract

Chemical warfare agents (CWAs) are one of the most toxic compounds. Degradation of CWAs using decontamination agents is one of the few ways to protect human health against the harmful effects of CWAs. A ferrate (VI)-based potential chemical warfare agent decontaminant was studied for the degradation of persistent nitrogen mustard (tris(2-chloroethyl)amine, HN3). By optimizing the reaction conditions, the complete degradation of HN3 was achieved in 4 min. The degradation products contained mostly reduced Fe species, which confirmed the environmental friendliness of the proposed decontamination solution.

## 1. Introduction

Chemical warfare agents (CWAs) are extremely toxic chemicals that are relatively easy to produce and that can be used against military personnel as a part of asymmetric warfare or against civilians by terrorist groups or non-state actors. An attempted murder using a Soviet-era Novichok class of chemical warfare nerve agent is one of the latest examples. Chemical warfare agents have been classified based on different properties [1]. Generally, the CW comprise six categories: lung insurance (choking agents), blood agents (cyanogens), vesicants (blister agents), nerve agents (anticholinesterase), incapacitants, and riot control agents (lacrimators) [2,3,4]. CWAs cause adverse health effects upon contact or by inhalation. Vesicants cause severe blistering of tissue, nerve agents inhibit the enzyme acetylcholinesterase, and blood agents cause tissue hypoxia [5,6,7].

Decontamination, contamination avoidance, and protection can reduce or remove the adverse health effects of CWAs if applied in time. Some chemical warfare agents and their decontamination methods can lead to toxic degradation by-products or have long degradation half-times [8]. Developing decontamination methods that completely and rapidly neutralise all chemical warfare agents without generating toxic residues is a complex challenge. To minimise toxic degradation processes and reduce degradation half-times, an ideal decontamination agent for chemical warfare agents should possess the following properties: broad-spectrum efficacy—it should be effective against a wide range of chemical warfare agents; rapid action—it should provide rapid decontamination to minimise exposure time and prevent further contamination; high efficiency—it should require minimal quantities to achieve effective decontamination, ensuring cost-effectiveness and practicality in real-world scenarios; non-damaging to surfaces—it should not soften or damage paints, coatings, polymeric seals, gaskets, or transparencies such as windscreens. In addition, the decontamination agent should have low toxicity and non-flammability, and it should have a minimal environmental impact, including biodegradability and low persistence in the environment [9].

Numerous decontamination methods have been reported so far, and the environmental degradation process of CWAs based on hydrolysis, oxidation, photolysis, and microbial degradation has been studied and reviewed [8,9,10,11,12,13]. Most of the work in the past has focused on hydrolysis and the oxidation-based degradation of CWAs [10,11,12,13,14,15]; additional decontamination strategies such as the use of non-photochemical or photochemical advanced oxidation process [16,17,18]; and heterogenous processes such as metal-organic frameworks [19,20,21,22], metal oxides [23,24,25,26], and polymers [27,28]. Direct energy application [29] or biotechnological [30] degradation may also be a viable option. In recent years, alternative decontamination strategies have been explored, such as non-photochemical or photochemical advanced oxidation processes and heterogeneous processes, including metal-organic frameworks, metal oxides, and polymers, as well as direct energy application or biotechnological degradation. Ferrate(VI) and nanoscale zero-valent iron are effective and environmentally friendly decontamination reagents for the destruction of CWAs [31]. Because of the relatively short half-times of degradation of the CWAs, as well as their good efficiency and low environmental impact, the use of ferrate(VI) can be promising method for the neutralization of CWA. In addition, its effectiveness against biological agents [32] could prove dual purpose of the ferrate(VI)-based decontamination methods.

The ferrate(VI) in our work was tested for the degradation of the chemical warfare agent HN3 (tris(2-chloroethyl)amine) under different conditions to optimize the effectiveness of the decontamination system. This research contributes to the development of more efficient and environmentally friendly decontamination strategies for CWAs. The persistent nitrogen mustard (tris(2-chloroethyl)amine, HN3) was selected as a model agent for its low solubility in water and relatively slow hydrolysis [8].

## 2. Materials and Methods

### 2.1. Preparation of Ferrate(VI)

Ferrate(VI) was prepared electrochemically in molten hydroxide according to the process described in detail elsewhere [33].

### 2.2. Preparation of Nitrogen Mustard HN3

“Caution! The nitrogen mustard is toxic chemical warfare agent. Because of its high toxicity, it was handled only by well-trained personnel using appropriate safety procedures in the accredited laboratory in compliance with Chemical Weapons Convention”.

**Figure 1:**
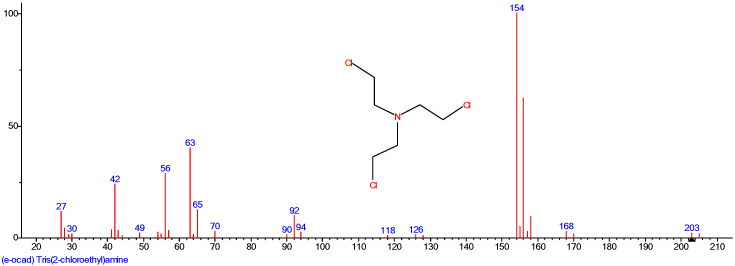
GC-MS analysis of the prepared HN3.

The nitrogen mustard HN3 was prepared in the Reference Chemical Laboratory of the Training and Testing Centre in Zemianske Kostolany. The laboratory is certified for the analysis of CWAs, and the production of CWA complies with the CWC. It was prepared by the reaction of triethanolamine with thionyl chloride in chloroform. The prepared by-product HN3 hydrochloride was treated with a solution of sodium carbonate in water. The resulting brownish oily product was separated by a separatory funnel and distilled. The product was then confirmed by GC-MS analysis, as shown in Figure 1, and its purity (99.8%) was verified by GC-FID.

### 2.3. Reaction of Ferrate Fe(VI) with Nitrogen Mustard HN3

To study the degradation rate of chemical warfare agent HN3 (tris(2-chloroethyl)amine), 0.7 mL of phosphate buffer solutions (sodium dihydrogen phosphate) with pH 3, 4, 5, and 6 were prepared in 4 mL glass vials. Then, 0.1 mL of a solution of HN3 in n-Hexane (200 ppm, 1.09 µM) mL was added to the buffer containing vials. Immediately, the addition of 0.2 mL freshly prepared ferrate(VI) solution in distilled water (1.09 mM) followed. After a selected time (2, 4, 8, 16 min), 250 µL of the reaction solution was transferred to 1 mL vials containing 500 µL of n-Hexane. The vials were shaken at 1000 RPM for 1 min [31]. After the organic layer separation, 200 µL of n-Hexane layer was collected, dried with sodium sulphate, and analysed using GC-FID to determine the concentration of remaining HN3.

## 3. Results and Discussion

### Degradation of Nitrogen Mustard

**Figure 2:**
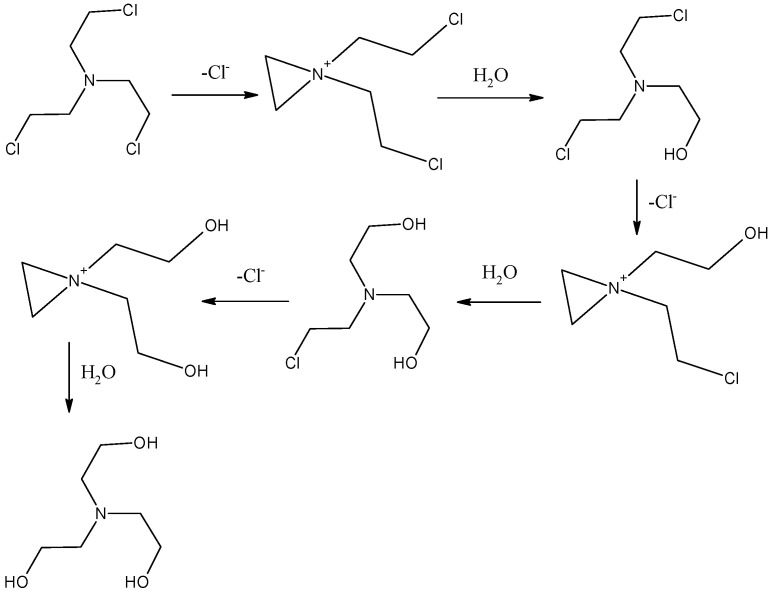
Hydrolysis mechanism of HN3 in water.

Since HN3 undergoes spontaneous hydrolysis in water [34] (Figure 2), at the beginning of our experiments, the hydrolysis the hydrolysis rate of the prepared HN3 was studied. The GC-FID analysis confirmed that hydrolysis of HN3 fulfilled the first-order kinetics equation [31]. Therefore, for analysing the kinetics data of HN3 hydrolysis, Equation (1) was used:

(1) $$c_{t}=c_{0}e^{-k_{1}t}$$

where *c_t_* denotes the residual concentration of HN3 in time *t*, and *c*_0_ stands for the initial concentration of HN3. The rate constant of the spontaneous hydrolysis of HN3 was determined to be 0.029 ± 0.008 min^−1^ at pH 6, which is in line with previously reported results.

**Figure 3:**
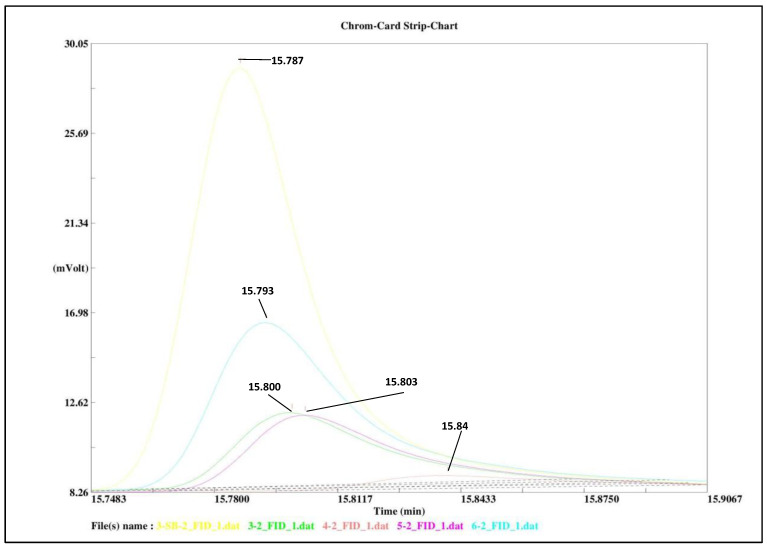
GC-FID chromatogram showing the remaining HN3 after 2 min of reactions with ferrate(VI) at different pH levels: 3-SB-2_FID (yellow)—hydrolysis of HN3 at pH 6, 3–2_FID (green)—reaction at pH 3, 4–2_FID (amber)—reaction at pH 4, 5–2_FID (purple)—reaction at pH 5, 6–2_FID (blue)—reaction at pH 6.

After these initial experiments, the oxidation power of ferrate(VI) toward HN3 was tested. The degradation of the nitrogen mustard HN3 was studied as a function of the concentration of remaining nitrogen mustard HN3 in a solution and time at various pH levels (Figure 3).

As expected, after the addition of purple ferrate Fe(VI) solution to a reaction mixture, the colour changed, and a brown precipitate containing iron(III)/iron(II) products as the final species was formed [26]. It can be supposed that oxidation of HN3 by ferrate(VI) is the 2e^−^ transfer step (Fe^VI^→Fe^IV^→Fe^II^) rather than the 1e^−^ transfer step, as suggested by previous reports concerning reactions of iron(VI) with amines [35,36].

**Figure 4:**
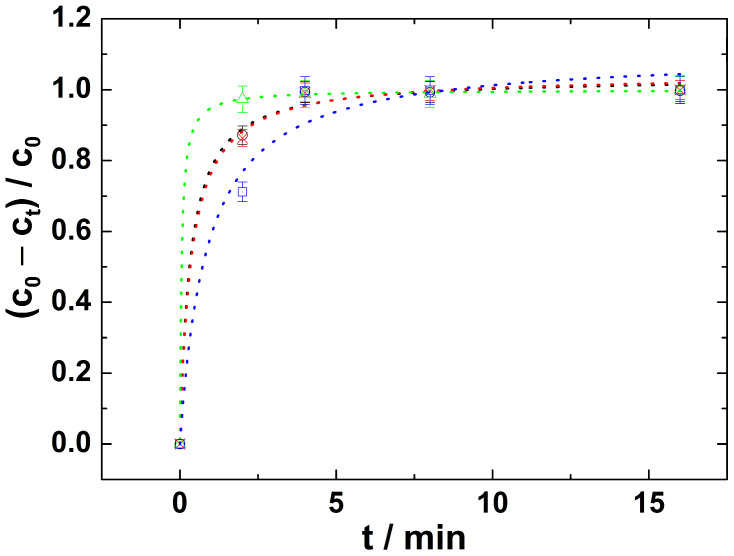
Kinetic curves representing time dependence of degradation of HN3 by Fe(VI) under different pH: circles are experimental data where the size of the individual points represents the standard deviations; lines are fitted curves according pseudo-second order kinetic Equation (2); blue colour—pH = 6; red colour—pH = 5; black colour—pH = 3; green colour—pH = 4.

The obtained data were studied using several kinetics models. Based on our measured kinetic data of the decomposition of HN3 using Fe(VI), it follows that the best fit was a pseudo-second order kinetics model. This observation is consistent with previous results, where the kinetics of amine oxidation by ferrate(VI) were monitored [35,36]. Therefore, the following equation was applied to calculate the second-order rate constant *k*_2_ for all degradation processes at all pH levels used:

(2) $$c_{t}=\frac{c_{e}^{2}k_{2}t}{1+c_{t}k_{2}t}$$

where *c_e_* is an equilibrium concentration of HN3. The rate of HN3 degradation is represented by kinetic curves in Figure 4.

As can be seen from Figure 3 the complete degradation of HN3 by ferrate(VI) was reached after approximately 4 min in all solutions of different pH levels. All curves that were calculated according to Equation (2) fit the experimental points well, while the individual statistical parameters are summarized in Table 1. This confirms the correctness of our reasoning that it is a reaction with pseudo-second order kinetics.

**Figure 5:**
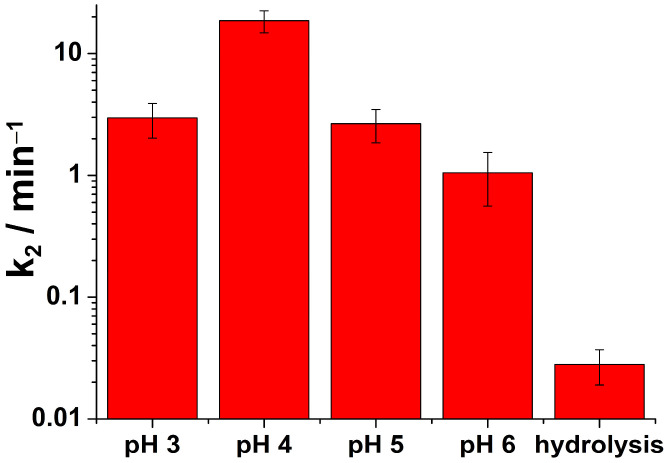
Calculated *k*_2_ rate constants of the degradation of HN3 by iron(VI) at different pH levels.

The rate of the HN3 oxidation using ferrate(VI) is much faster as the rate of HN3 hydrolysis (25 h) [14], and faster than decontamination of other persistent vesicant sulphur mustard using TiO_2_ (24 h) [37], sulphur-doped TiO_2_ (2 h) [38], or MgAl_2_O_4_ (4 h) [39], as an example. Individual rate constants obtained in our experiments are shown in Figure 5.

In Figure 5, the bar diagrams of calculated rate constants *k*_2_ according to Equations (1) and (2) at different pH levels are plotted.

From Figure 5, it is easily visible that the decomposition rate of HN3 by ferrate(VI) strongly depends on the pH of the solution. The highest rate was observed for pH = 4, followed by pH = 5 and pH = 3. The lowest rate was observed for the pH = 6. The lower decomposition rate of nitrogen mustard at pH = 3 compared to pH = 4 was probably caused by the strong self-decomposition reaction of ferrate(VI) to Fe(III)/Fe(II) by water via the intermediary of Fe(IV) and Fe(V) species, which could have reduced the efficiency of the degradation process itself. At pH = 3, H_2_FeO_4_ was mainly present due to the pKa = 3.5 ± 0.2 [40,41]. At this acidic pH, the formation of a diferrate(VI) with fast intramolecular oxo-coupling producing O_2_ and diferryl(IV) species was proposed. The authors of this study point out that in the acidic solutions, the kinetics of ferrate(VI)-mediated water oxidation (ferrate decomposition) becomes a complex problem. Similarly to our study, a second-order decay process can be resolved with HN3. However, the self-decomposition of ferrate(VI) is faster than the oxidation of nitrogen mustard with condensation and dimerization of monomeric ferrate as the rate-determining step [40]. It is evident that degradation by ferrate(VI) is significantly faster at all pH levels than hydrolysis alone, which is one of the most important parameters under combat conditions.

In Table 1, the calculated values of all rate constants are summarised.

It can be easily seen that the rate of the decomposition of HN3 by ferrate(VI) was at the same pH levels approximately two orders of magnitude higher than that of spontaneous hydrolysis of HN3. This means that the impact of hydrolysis on the decomposition of HN3 is very low compare to ferrate(VI) and can be neglected during calculations. In addition, this means that Fe(VI) is a strong enough oxidizing agent against nitrogen mustard even at an almost neutral pH, which, in field conditions, means the simplification of handling during the eventual decontamination of surfaces, people, vehicles, etc. using ferrate(VI) solution.

## 4. Conclusions

This study aimed to test a potentially fast-acting, highly efficient and environmentally friendly decontamination agent based on ferrate Fe(VI) for its ability to degrade the persistent chemical warfare agent HN3. The potential of the Ferrate(VI)-based decontamination agents also lies with its relative ease of use, potential effectiveness against biological agents, and non-damaging and non-flammable properties. Our research focused on identifying the optimal conditions for degradation, with particular attention paid to reaction rates under different pH levels to achieve the fastest and most effective degradation.

Our experiments revealed that the reaction rate was fastest at a lower pH of 3. However, the degradation of the nitrogen mustard HN3 was nearly 100% within just four minutes across the full range of pH levels tested. This remarkable result demonstrates the efficiency and effectiveness of ferrate Fe(VI) as a promising candidate for the decontamination of persistent chemical warfare agents.

Furthermore, the formation of Fe(III) and Fe(II) species as ferrate reduction products is acceptable from an environmental perspective, as these products pose no significant harm to the environment.

Our future work will focus on the degradation of other persistent chemical warfare agents and optimising the degradation conditions for practical applications of Fe(VI)-based decontamination agents. These efforts hold great promise for the development of safer, more efficient, and environmentally friendly solutions for the decontamination of hazardous materials.

## Figures and Tables

**Table 1:** The rate constants of oxidation of the HN3 by Fe(VI) at different pH levels.

pH	*k*_2_ (min^−1^)	χ^2^	Adjusted R-Squared
3	2.961 ± 0.942	6.48 × 10^−4^	0.997
4	18.596 ± 3.750	1.16 × 10^−5^	0.999
5	2.658 ± 0.808	6.87 × 10^−4^	0.996
6	1.054 ± 0.498	4.59 × 10^−3^	0.975

## Data Availability

The data presented and analyzed in this study are available on reason- able request from the corresponding author.
